# Epigenetic regulation of angiogenesis and its therapeutics

**DOI:** 10.1186/s44342-025-00038-3

**Published:** 2025-02-11

**Authors:** Dong Kyu Choi

**Affiliations:** BK21 FOUR KNU Creative BioResearch Group, School of Life Science and Biotechnology, Kyungpook National University, Daegu, Korea

**Keywords:** Angiogenesis, Epigenetic modification, Epigenetic drugs

## Abstract

Angiogenesis, the formation of new blood vessels from preexisting ones, is essential for normal development, wound healing, and tissue repair. However, dysregulated angiogenesis is implicated in various pathological conditions, including cancer, diabetic retinopathy, and atherosclerosis. Epigenetic modifications, including DNA methylation, histone modification, and noncoding RNAs (e.g., miRNAs), play a crucial role in regulating angiogenic gene expression without altering the underlying DNA sequence. These modifications tightly regulate the balance between pro-angiogenic and anti-angiogenic factors, thereby influencing endothelial cell proliferation, migration, and tube formation. In recent years, epigenetic drugs, such as DNA methyltransferase inhibitors (e.g., azacitidine, decitabine), histone deacetylase inhibitors (e.g., vorinostat, romidepsin), and BET inhibitors (e.g., JQ1), have emerged as promising therapeutic strategies for targeting abnormal angiogenesis. These agents modulate gene expression patterns, reactivating silenced tumor suppressor genes while downregulating pro-angiogenic signaling pathways. Additionally, miRNA modulators, such as MRG-110 and MRG-201, provide precise regulation of angiogenesis-related pathways, demonstrating significant therapeutic potential in preclinical models. This review underscores the intricate interplay between epigenetic regulation and angiogenesis, highlighting key mechanisms and therapeutic applications. Advancing our understanding of these processes will enable the development of more effective and targeted epigenetic therapies for angiogenesis-related diseases, paving the way for innovative clinical interventions.

## Introduction

Angiogenesis, the process through which new blood vessels form from preexisting ones, is crucial for growth, development, and wound healing. This process is orchestrated through an intricate interplay of molecular and cellular events that stimulate endothelial cells (ECs) to proliferate, migrate, and eventually form new capillary networks [1]. The term “angiogenesis” itself was first introduced by the Scottish surgeon John Hunter, but the study of how blood vessels develop did not gain substantial attention until Judah Folkman proposed in the 1970s that angiogenesis was pivotal to tumor growth, suggesting that inhibiting angiogenesis could serve as a potential cancer therapy strategy [2].

Historically, the understanding of angiogenesis has evolved from merely recognizing its role in normal physiology to acknowledging its critical involvement in a host of diseases. Beyond its essential functions in embryonic development and wound healing, angiogenesis is now known to play a significant role in the pathology of diseases such as cancer, diabetic retinopathy, rheumatoid arthritis, age-related macular degeneration (AMD), and atherosclerosis. In the context of cancer, for instance, the growth and spread of tumors are heavily dependent on the supply of nutrients and oxygen through blood vessels. Hence, tumors can stimulate the pro-angiogenic switch to support their growth [3].

The control mechanisms of angiogenesis are tightly regulated at the genetic, proteomic, and epigenetic levels in various diseases. In particular, epigenetic modifications, including DNA methylation, histone modification, chromatin remodeling, and miRNAs, regulate genes associated with tumor progression and angiogenesis by either activating or inhibiting their expression. For this reason, epigenetic modifications have emerged as promising therapeutic targets, with some epigenetic drugs currently being developed for the treatment of multiple diseases [4].

In this review, we will explore into the mechanisms driving angiogenesis, with a particular focus on the role of epigenetic modifications in regulating this process. Furthermore, we will highlight recent advancements in identifying therapeutic targets and evaluate the clinical and preclinical potential of epigenetic drugs for treating angiogenesis-related diseases.

## Pathological angiogenesis

The development of the vascular system during embryogenesis occurs in several stages. First, blood vessels are formed from mesodermal progenitors, known as angioblasts, which differentiate into ECs and form primitive blood vessels. After vasculogenesis, new vessels sprout from preexisting ones and mature through processes such as branching, elongation, and capillary network formation, which are essential for subsequent tissue development. Next, vessels undergo remodeling, involving pruning of unnecessary vessels and strengthening others to form a mature vascular network that meets the metabolic demands of various tissues. Finally, arteries and veins were specified, a process influenced by flow dynamics and endothelial signaling including ephrin-B2 (arterial marker) and Eph-B4 (venous marker) [5].

**Fig. 1:**
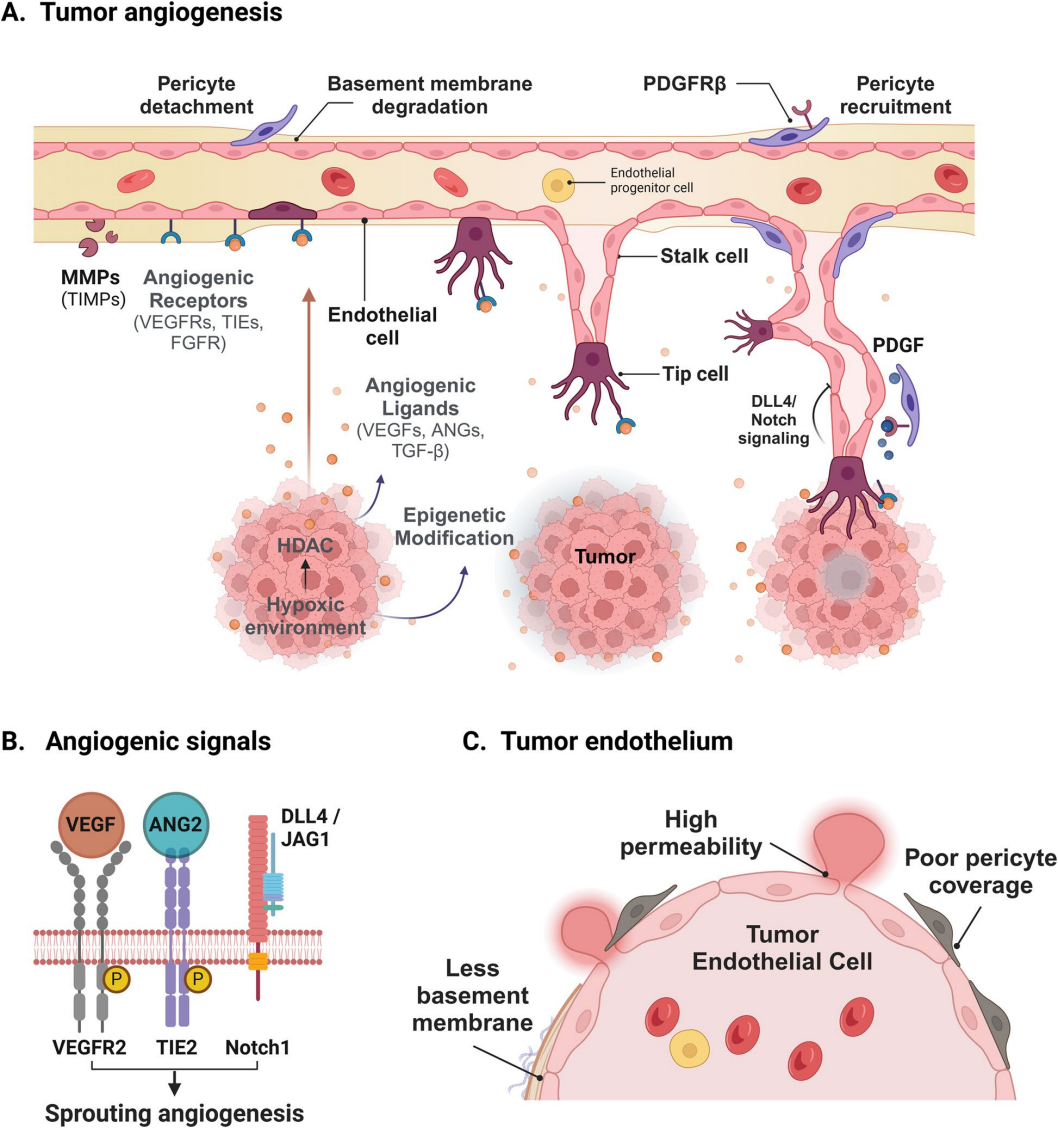
Tumor angiogenesis and vascular abnormalities.** A** Schematic representation of the angiogenic process in tumor progression highlighting pericyte detachment, basement membrane degradation, and endothelial cell migration influenced by angiogenic factors (VEGFs, ANGs, TGF-β) in a hypoxic environment. Tip and stalk cells differentiation is regulated by DLL4/Notch signaling, with pericyte recruitment mediated by PDGFRβ. **B** VEGFR, TIE2, and Notch1 signaling pathways promoting sprouting angiogenesis. **C** Abnormal tumor vasculature, characterized by poor pericyte coverage, decreased basement membrane, and high vessel permeability. Figure was created with BioRender.com

Under physiological conditions, ECs remain in a quiescent state. However, they retain remarkable plasticity, enabling them to respond dynamically to pathological stimuli. A notable example is tumor angiogenesis, where various pro-angiogenic signals precisely regulate the formation, proliferation, and migration of blood vessels [1]. In tumors, ECs migrate following basement membrane degradation to maintain their basal-luminal polarity. After endothelial growth, the basement membrane is redeposited for coverage [6]. Thus, ECs sprout, forming a tip cell at the proliferating end. Vascular endothelial growth factor (VEGF), highly expressed in tumor cells, activates VEGFR2 signaling in tip cells to enhance glycolysis for energy production and upregulates delta-like 4 (DLL4), which signals to stalk cells [7]. Signaling activity of Notch is low in proliferating tip cells where DLL4 and VEGFR2 are highly expressed [8]. In contrast with tip cells, stalk cells possess high Notch signaling activity by downregulating VEGFR2 and NRP1 while upregulating VEGFR1 [9]. Furthermore, Notch activity in stalk cells is maintained by JAG1-mediated antagonism of DLL4 [10]. Interestingly, Notch inhibition enhances angiogenic sprouting in tumors. However, the resulting ECs exhibit disrupted vascular hierarchy and unstable junctional complexes, ultimately leading to reduced tumor growth [11] (Fig. 1A).

Several key molecular pathways drive angiogenesis, orchestrating the complex interactions between EC types, support cells (like pericytes), and the extracellular matrix. Among the most prominent are VEGF and its receptors. VEGF binds to specific receptors on ECs, triggering a cascade of signaling events that promote their survival, migration, and proliferation [11]. Through VEGFR2 binding, VEGF-A promotes the growth of new blood vessels and their migration, while VEGFR1 acts as an endothelial decoy receptor, negatively regulating blood vessel morphogenesis [12]. Tumor cells also utilize VEGF for angiogenesis. However, VEGF-induced tumor vessels exhibit leaky vasculature characterized by poor pericyte coverage and a thin basement membrane, which hinders chemotherapeutic agent delivery [13]. For this reason, targeting VEGF and other components to normalize tumor vasculature has emerged as a promising therapeutic approach beyond cancer, and VEGF also plays a critical role in the pathogenesis of AMD, where overgrowth of blood vessels in the eye leads to vision loss, thereby VEGF-targeting antibodies such as ranibizumab and bevacizumab have demonstrated significant therapeutic efficacy in ADM treatment [14]. Especially, VEGFR-3 is primarily involved in the regulation of lymphangiogenesis, the formation of lymphatic vessels, which is important for maintaining fluid balance and immune function.

Another key molecular pathway is angiopoietins and tie receptors. Tie receptors, also known as TEK, belong to the receptor tyrosine kinase family and are crucial for later stages of vascular development and vascular stability. Angiopoietin-1 (ANG1) is the primary agonistic ligand of TIE2. When ANG1 binds to TIE2, it induces receptor dimerization and autophosphorylation, leading to the activation of downstream signaling via AKT, which inhibits the transcription factor forkhead box protein O1(FOXO1), thus promoting vascular stability and EC survival. This signaling supports the recruitment of pericytes and smooth muscle cells to newly formed blood vessels and maintains the quiescence of blood vessels (Fig. 1B).

Angiopoietin-2 (ANG2) functions as a context-dependent agonist and antagonist for the TIE2 receptor. ANG2 is expressed in ECs and stored in WP bodies. During inflammation, ANG2 is secreted by ECs and suppresses ANG1-TIE2 signaling. In addition, ANG2 destabilizes endothelial monolayers by actin stress fiber formation. In tumors, ANG2 levels are often elevated, which disrupts the balance between ANG1 and ANG2, leading to abnormal blood vessel formation and increased permeability. In addition, abnormal TIE2 signaling is implicated in various vascular diseases, including diabetic retinopathy and atherosclerosis. Therefore, targeting the TIE2 pathway, particularly by inhibiting ANG2 or enhancing ANG1 signaling, is a strategy being explored in cancer therapy through normalization of tumor blood vessels. Therapeutic modulation of this pathway holds potential for treating these conditions by restoring vascular stability and preventing excessive angiogenesis or vessel leakage [15].

Furthermore, genetics, proteomics, and epigenetic changes in these key molecules result in the activation of pro-angiogenic signals and cause dysregulated angiogenesis. In pathological conditions, pro-angiogenic signals such as VEGF and angiopoietins dominate anti-angiogenic signals to induce neovascularization through sprouting angiogenesis, intussusceptive angiogenesis, vascular mimicry, and recruitment of EC progenitors [16]. In tumors, abnormal vasculature which is characterized by small vessel diameter, heterogeneous vascular density, and high permeability is formed due to dysfunction in pericytes and base basement membrane integrity [17, 18] (Fig. 1C).

Understanding epigenetic changes in angiogenic key molecules provides insights how the vascular system is regulated during normal and pathological conditions. Moreover, elucidating these pathways offers potential therapeutic targets for modulating angiogenesis in adult diseases, where similar molecular mechanisms are often reactivated.

## Epigenetic regulation of angiogenesis

**Fig. 2:**
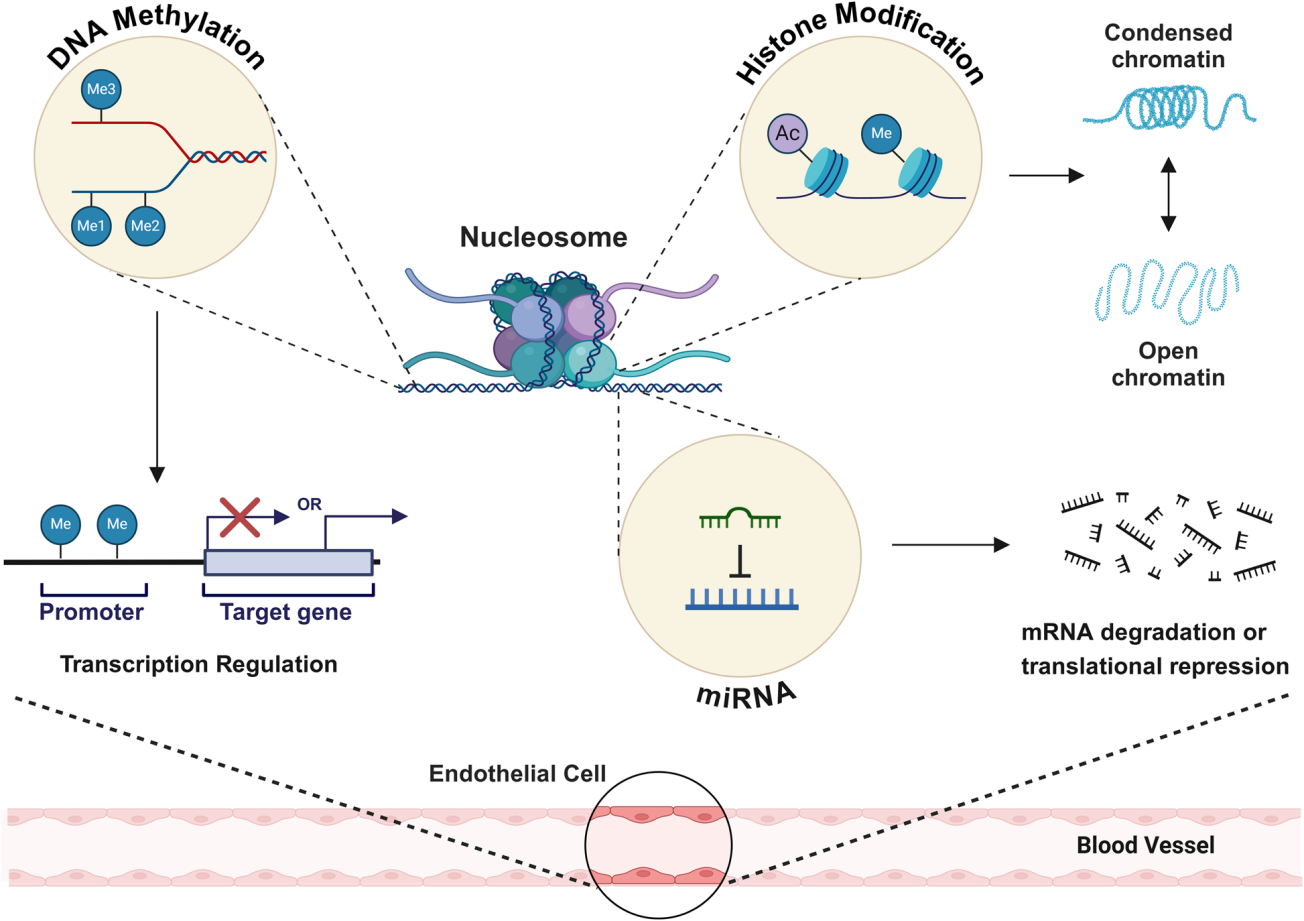
Epigenetic regulations in ECs. Diagram illustrating the key mechanisms of epigenetic regulation in ECs, including DNA methylation, histone modification, and miRNA. DNA methylation at promoter regions can result in transcriptional repression of target genes. Histone modifications, such as acetylation and methylation, alter chromatin structure, dynamically switching between open and condensed states, which affects gene expression. miRNAs contribute to posttranscriptional regulation by binding to mRNA leading to either degradation or translational repression. Figure was created with BioRender.com

Epigenetic regulation refers to the process by which gene expression is controlled without altering the underlying DNA sequence. Instead of changing the genetic code itself, epigenetic regulation modifies the DNA or associated proteins (such as histones) through mechanisms like DNA methylation, histone modifications, and noncoding RNAs, which can regulate the gene expression or modulate their activities [19]. Therefore, understanding epigenetic alterations in physiological and pathological events is important for identifying therapeutic target and improving diagnostic approaches [20] (Fig. 2).

DNA methylation, the addition of methyl groups to the DNA molecule, typically at cytosine bases in CpG islands, is implicated in gene repression by blocking the binding of transcription factor; however, in some case, it is also associated with gene activation [21]. This DNA methylation is closely related to onset and progression of disease [22]. In breast tumors, CpGs in upstream promoter regions are hypermethylated, while CpGs in the gene body are hypomethylated [23]. Tumors utilize angiogenic signals for their sustained growth through the hypermethylation of anti-angiogenic factors. For example, hypermethylation of THBS-1, an angiogenesis inhibitor with tumor suppressor properties, has been observed in various cancers. Furthermore, these epigenetic changes in THBS-1 are associated with vascular invasion [24] and impaired TGF-β signaling [25].

Under pathological conditions characterized by high TGF-β2 levels, ECs undergo endothelial-to-mesenchymal transition (EndMT), a process well documented in tumors and atherosclerosis. Recent single-cell sequencing revealed that ECs exhibit plasticity with transient mesenchymal gene expression following myocardial infarction. Interestingly, this plasticity is reversible and associated with changes in DNA methylation at regulatory regions [26]. Moreover, blood flow epigenetically regulates angiogenic gene expression by modulating genome-wide DNA methylation in a DNA methyltransferase (DNMT)-dependent manner. In mouse and HUVEC models, shear stress induces DNMT expression, while a reduction of DNMT levels with azacitidine or siRNA significantly decreases shear stress-induced endothelial inflammation and lesion formation in atherosclerosis. These findings suggest that disturbed blood flow alters endothelial gene expression and promotes atherosclerosis through DNMT-dependent DNA hypermethylation, highlighting DNMT as a promising therapeutic target for atherosclerosis [27].

In addition, hypermethylation of tissue inhibitors of metalloproteinase-2 (TIMP-2) and TIMP-3 has been observed in various tumors [28]. TIMP is a family of proteins that regulate the activity of matrix metalloproteinase (MMP), enzymes responsible for extracellular matrix (ECM) degradation. By inhibiting MMP-2, which plays a crucial role in ECM degradation, TIMP-2 can prevent the breakdown of the ECM, a critical step in angiogenesis. Meanwhile, TIMP-3 inhibits ADAMs, including ADAM17. This inhibition affects ECM degradation and also the shedding of membrane-bound precursors of growth factors and cytokines that are important for angiogenesis. TIMP3 also blocks VEGF binding to VEGFR2 and inhibits downstream angiogenic signaling [29]. Hypoxia also enhances HDAC activity. Under hypoxic conditions, HDAC1 is induced, and this overexpression negatively regulates the expression of p53 and von Hippel-Lindau (VHL) tumor suppressor gene, thereby stimulating angiogenesis. Patient with VHL disease possesses mutations in VHL gene, resulting in the accumulation of HIF-1α in cells and the constitutive activation of angiogenic genes, including VEGF, PDGF-B, and TFG-α. Notably, additional hypermethylation of tumor suppressor genes causes the inactivation of VHL alleles, leading to VHL-associated hemangiomas, highlighting the potential role of epigenetic modification, particularly HDAC inhibitors, in disease progression [30]. Similarly, when trichostatin A (TSA), a specific HDAC inhibitor, is applied, the expression of HIF-1α and VEGF is downregulated in vitro, and in vivo angiogenesis is blocked [31]. Interestingly, the epigenetic regulation of lymphangiogenesis has been reported. In gastric cancer cell lines, demethylation-induced VEGF-C expression, which correlates with lymphatic vessel density and metastasis, has been observed [32].

Eukaryotic DNA is wrapped around histone octamers, forming nucleosomes to maintain a highly ordered chromatin structure. Chemical modifications such as acetylation, methylation, and phosphorylation of histone proteins can influence chromatin structure, making it either more or less accessible for transcription. Generally, HAT-mediated acetylation increases gene transcription, whereas HDAC-mediated deacetylation inhibits transcription [33]. During angiogenesis, diverse histone methyltransferases have been reported to promote proliferation, invasion, and spouting of EC, and their expression is closely associated with poor prognosis. HDAC7 is expressed in the vascular endothelium during development. A mouse model lacking HDAC7 showed embryonic lethality due to decreased endothelial cell adhesion following vascular dilatation and rupture [34]. In addition, its silencing leads to increased PDGFB expression and decreased EC migration [35]. Furthermore, VEGF-mediated upregulation of ANG2, survivin, and CXCR4 was inhibited via HDAC inhibition [36].

Blood vessels also express class III HDACs, known as sirtuins. Among them, SIRT1, nicotinamide adenine dinucleotide (NAD +)-dependent histone deacetylase, plays a key role in angiogenesis. Loss of SIRT1 downregulates vascular remodeling genes and reduces angiogenic sprouting. Additionally, SIRT1 deacetylates the forkhead transcription factor O1 (FOXO1), a negative regulator of vessel development, thereby restraining its anti-angiogenic effect [37]. Caloric restriction (CR) elevates NAD + levels, modulating the expression and activity of sirtuin family. Interestingly, CR restores vascular EC functions in aged mice by increasing arterial SIRT1 expression and nitric oxide (NO) bioavailability compared to ad libitum-fed counterparts [38]. Through interacting with SIRT1, CR also modulates the activity of key angiogenesis-related pathways, including AMPK, mTOR, and IGF-1. By activating AMPK, SIRT2 expert protective effects on age-related vascular remodeling. These findings suggest that sirtuin activation via CR may serve as a promising strategy for preventing or treating cardiovascular diseases.

*Urbich* and colleagues reported that suppression of MLL, a histone lysine 4 methyltransferase, significantly decreased migration and sprout formation in HUVEC cells, indicating the requirement of methyltransferase activity in EC functions [39]. MLL regulates angiogenesis not only by methylating lysine residue but also by recruiting MOF [40]. SETD8/KMT5A is a histone lysine methyltransferase known for the mono-methylation of histone H4 lysine 20 [41]. It participates in cell cycle progression, and its suppression leads to cell cycle defects [42]. SETD8 regulates angiogenesis through an osteopontin-dependent mechanism. Pharmacological inhibition of SETD8 not only inhibits the differentiation of human-induced pluripotent stem cell into endothelial cell lineage but also rescues abnormal pathological angiogenesis in retina of an oxygen-induced retinopathy mouse model [43].

MicroRNAs (miRNAs) are highly expressed in ECs and play a significant role in the regulation of angiogenesis. miRNAs are small, noncoding RNAs that typically function by binding to complementary sequences on target messenger RNAs, leading to their degradation or inhibition of translation [44]. In the context of angiogenesis, several miRNAs have been identified as critical regulators, influencing the expression of genes that control endothelial cell function, vascular stability, and the formation of new blood vessels.

Mice with vascular-specific Dicer deletion show impaired angiogenesis and alteration in angiogenesis-related gene [45, 46]. Let-7 and miR-103/107 are hypoxia-responsive miRNAs and are strongly expressed in ECs. These miRNAs target Argonaute1 (AGO1), a critical component of the microRNA-induced silencing complex (miRISC) necessary for the silencing of VEGF mRNA, thereby leading to the translational derepression of VEGF. Notably, Let7b promotes EC proliferation and motility by targeting TIMP-1 within the endothelium [47]. miR-210, also known as “hypoxia-miRNA,” is upregulated under hypoxic conditions. By targeting ephrin-A3, negative regulator, miR-210 promotes angiogenesis [48, 49]. miR-126 is one of the most studied miRNAs in angiogenesis, and miR-126 is primarily expressed in ECs and promotes vascular development by enhancing the signaling pathways of VEGF and fibroblast growth factor (FGF). Moreover, it targets negative regulators of the VEGF pathway, such as SPRED1 and PIK3R2, thus promoting endothelial cell proliferation and migration [50]. By targeting p120RasGAP, a negative regulator of the Ras signaling pathway, miR-132 promotes the angiogenic switch in ECs. Thus, miR-132 enhances Ras signaling, leading to increased endothelial cell proliferation and new blood vessel formation [51].

Some miRNAs inhibit angiogenesis by targeting pro-angiogenic genes or signaling pathways. Circulating miR-15b inhibits angiogenesis by directly targeting the 3′-UTR of VEGF. In proliferative diabetic retinopathy patients, Yang et al. found significant inverse relationship between low levels of miR-15b and high level of VEGF [52]. Their overexpression also has been shown to suppress tumor growth by limiting the blood supply to the tumor [53]. miR-221/222 inhibit angiogenesis by targeting the c-Kit receptor, a key player in endothelial cell migration and survival. By downregulating c-Kit, miR-221/222 reduce the angiogenic capacity of ECs, thereby contributing to the inhibition of abnormal blood vessel formation [54]. miR-29b targets several components of angiogenesis including VEGF-A, ANPTL4, PDGF, and MMP9. Through its regulation of collagen remodeling and angiogenic signaling, miR-29b exerts an anti-angiogenic effect and reduces fibrillar collagen synthesis in a murine orthotopic breast cancer model [55]. Moreover, miR-424 regulates angiogenesis via targeting VEGFR2 and fibroblast growth factor receptor 1 (FGFR1), thereby suppressing EC proliferation, migration, and tube formation. However, miR-424 promoted angiogenesis in hypoxic conditions, highlighting its context-dependent effect [56].

## Epigenetic drugs

**Fig. 3:**
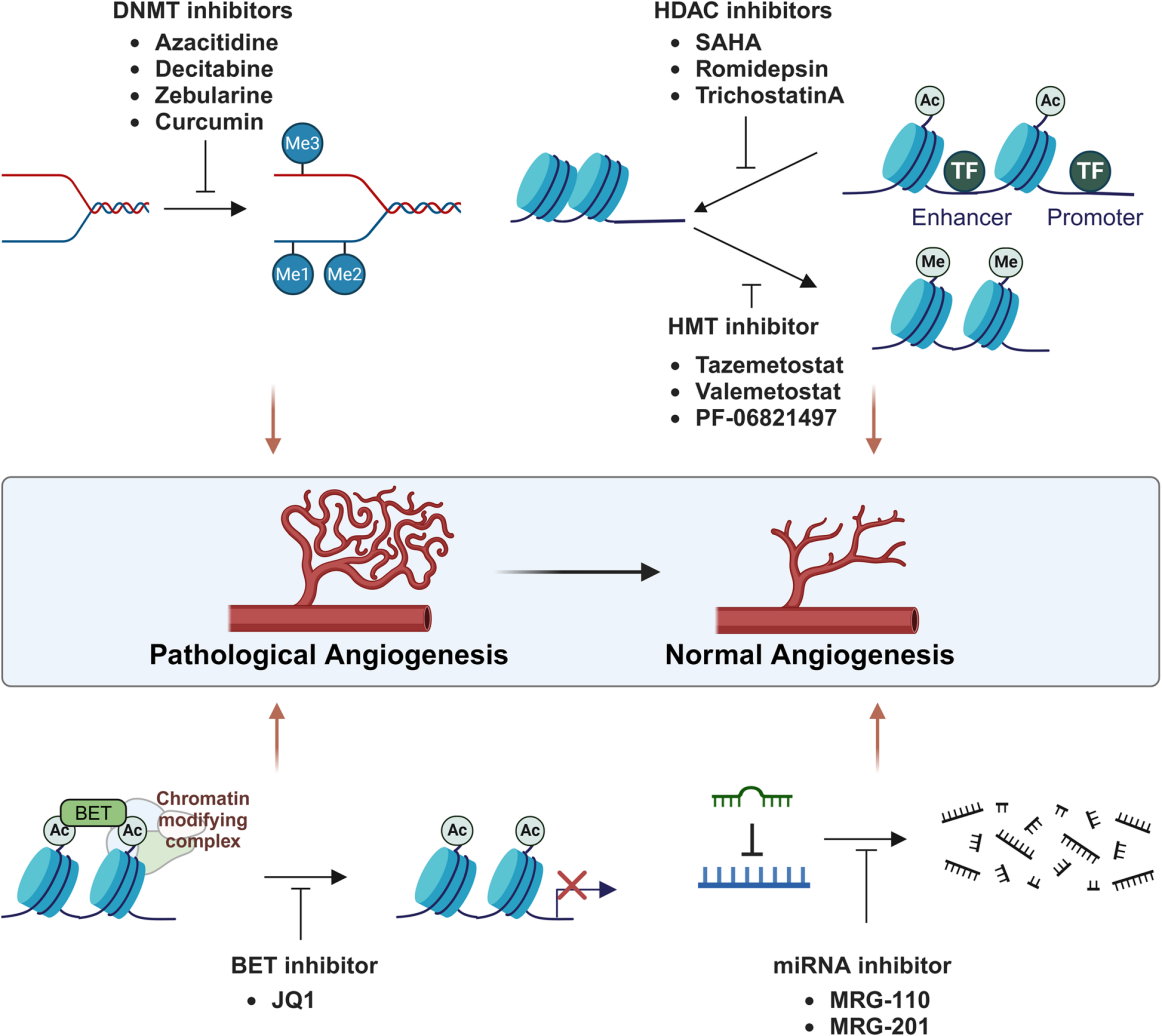
Therapeutic targets in epigenetic modification of angiogenesis. Schematic representation illustrating the role of various epigenetic inhibitors in regulating pathological angiogenesis. DNMT inhibitors (e.g., azacitidine, decitabine, zebularine, curcumin) prevent DNA methylation, while HDAC inhibitors (e.g., SAHA, romidepsin, trichostatin A) and HMT inhibitors (e.g., tazemetostat, valemetostat, PF-06821497) alter histone acetylation and methylation, leading to modifications in chromatin structure and gene expression. BET inhibitors, such as JQ1, disrupt chromatin-modifying complexes, and miRNA inhibitors (e.g., MRG-110, MRG-201) interfere with miRNA-mediated mRNA degradation. Collectively, these therapeutic interventions aim to reduce or normalize angiogenesis, underscoring their potential in treating angiogenesis-related diseases. Figure was created with BioRender.com

Given the crucial role of epigenetics in angiogenic gene regulation, epigenetics represents potential target for diseases characterized by abnormal vessel growth, such as cancer, diabetic retinopathy, and AMD (Fig. 3).

DNMT inhibitors reactivate silenced anti-angiogenic genes or downregulate pro-angiogenic genes. Azacitidine (Vidaza) is a hypomethylating agent and was first tested for the treatment of myelodysplastic syndrome decades ago, with the first large randomized trial published in 2002 [57]. It is also being explored for its anti-angiogenic effects in glioblastomas. In patient-derived xenograft models, highly vascularized tumors responded to azacitidine treatment, whereas poorly vascularized xenografts showed no response. Furthermore, glioblastomas treated with azacytidine showed significantly reduced angiogenesis [58]. Decitabine (Dacogen), another DNMT inhibitor with similar effects, is being investigated in various cancers, including solid tumors, to assess its impact on angiogenesis through epigenetic modulation [59].

HDAC inhibitors lead to the accumulation of acetylated histones, which generally results in an open chromatin structure and increased gene transcription. HDAC inhibitors can upregulate anti-angiogenic factors or downregulate pro-angiogenic factors. Vorinostat (SAHA), an FDA-approved HDAC inhibitor for cutaneous T-cell lymphoma, is under investigation for its potential to inhibit angiogenesis by downregulating VEGF and other pro-angiogenic factors in human umbilical vein endothelial cell (HUVEC). Additionally, SAHA upregulated the expression of VEGF competitor and semaphorin III at both mRNA and protein levels [60]. Interestingly, SAHA also directly repressed the promoter activity of VEGF-C in a human breast cancer cell line, thereby inhibiting lymphangiogenesis [61].

Romidepsin (Istodax), another HDAC inhibitor approved for T-cell lymphoma, is being studied for its anti-angiogenic properties in solid tumors. In neuroblastomas, high expression of VEGF and the extent of angiogenesis are associated with poor prognosis, and romidepsin inhibits angiogenesis by downregulating VEGF expression [62, 63]. In a subcutaneous xenograft hepatocellular carcinoma model, panobinostat, approved for multiple myeloma, significantly delayed tumor growth and resulted in prolonged overall survival with reduced angiogenesis [64]. These findings suggest a potential role for HDAC inhibitors as anti-tumor and anti-angiogenic agents, particularly through the modulation of VEGF signaling.

Bromodomains play a critical role in epigenetics by reading and interpreting the histone code. Specifically, bromodomains recognize and bind to acetylated lysine residues on histone tails. This binding facilitates the recruitment of chromatin-modifying complexes and transcriptional machinery to specific genome regions, thereby regulating gene expression [65]. For that reason, several molecules targeting bromodomains within the BET family (bromodomain and extra-terminal domain) have been tested in early clinical trials. The well-known BET inhibitor JQ1 reduces angiogenesis by downregulating key pro-angiogenic factors, such as c-Myc and VEGF. JQ1 specifically targets BRD4, a protein that binds to acetylated lysine residues on histones. By inhibiting BRD4, JQ1 disrupts its ability to recruit P-TEFb, a critical factor in the transcriptional elongation, thereby inhibiting the transcription of angiogenesis-promoting genes. In multiple myeloma, JQ1 also inhibits the interaction between dysregulated BRD4 and igG enhancers within the MYC locus, suppressing MYC and its downstream signals [66]. OTX015 (MK-8628), a BET inhibitor in early clinical trials for glioblastoma and hematologic malignancies, has demonstrated potential anti-angiogenic effects. It modulates the expression of angiogenesis-related genes, such as VEGF-A and CD31, in vitro and decreases microvessel density in ependymoma xenograft models [67].

LSD1 (lysine-specific demethylase 1) inhibitor blocks histone demethylation, thereby affecting the expression of genes involved in angiogenesis. LSD1 overexpression has been observed in multiple cancer types and plays a role in regulating genes associated with cell proliferation and survival, highlighting it as a promising target for therapeutic interventions [68]. GSK2879552 is an irreversible LSD1 inhibitor that plays a critical role in gene regulation by demethylating histones. This inhibition affects the expression of genes involved in various cellular processes such as proliferation and differentiation [69]. While GSK2879552 has shown promise in preclinical models of small cell lung cancer (SCLC) and acute myeloid leukemia (AML), its connection to angiogenesis remains indirect. LSD1, the target of GSK2879552, regulates gene expression pathways that can influence tumor microenvironments, including those related to angiogenesis. For instance, LSD1 demethylates HIF-1α at lysine 391 residues, which protects HIF-1α form ubiquitin-mediated protein degradation [70]. By inhibiting LSD1, GSK2879552 may alter the tumor microenvironment and influence angiogenesis, although this effect has been less directly studied compared to its impact on tumor cell proliferation. ORY-1001 (iadademstat) is a potent and selective covalent LSD1 inhibitor currently in clinical trials for acute myeloid leukemia and small cell lung carcinoma. Preclinical evidence suggests that ORY-1001 may inhibit angiogenesis through epigenetic mechanisms via LSD1 inhibition [71].

Histone methyltransferase (HMT) inhibitors are gaining significant attention as promising therapeutic targets for cancer and other diseases. These inhibitors target enzymes that add methyl groups to histones, a key modification influencing gene expression. By inhibiting these enzymes, HMT inhibitors can reactivate silenced tumor suppressor genes or suppress oncogenes, making them a valuable strategy in cancer therapy [69]. One of the most important HMTs in tumors is enhancer of zeste homolog 2 (EZH2), a component of the polycomb repressive complex 2 (PRC2). EZH and EZH2 induce trimethylation of histone H3 at lysine 27 (H3K27Me3). EZH2 is highly overexpressed in prostate cancer, colorectal cancer, and several other cancers and promotes carcinogenesis by repressing tumor suppressor genes, such as p21 and p16. Interestingly, mutations in the catalytic SET domain of EZH2 have been observed in diffuse large B-cell lymphomas and follicular lymphomas, contributing to oncogenic potential of EZH2.

Recently, two EZH inhibitors were approved by FDA. Tazemetostat (Tazverik), an EZH2-specific small molecule inhibitor, was approved for epithelioid sarcoma and follicular lymphoma. It is also under investigation for its potential to inhibit angiogenesis by modulating the expression of genes regulated by histone methylation. This aligns with the observation that EZH2 epigenetically regulates the expression of VASH1 and controls tumor angiogenesis in various types of cancer [72], demonstrating promising role of EZH2 inhibitors in various disease [73]. Valemetostat, an oral dual inhibitor targeting both wild-type and mutated forms of EZH2 and EZH1, has been approved for the treatment of relapsed or refractory adult T-cell leukemia/lymphoma (r/r ATL). Furthermore, clinical trials of investigating valemetostat for non-Hodgkin lymphoma (phase I, NCT02732275) and r/r ATL (phase II, NCT04102150) are currently ongoing. PF-06821497 (mevrometostat) effectively inhibits the EZH2 Y641N mutation and is currently being evaluated in a phase III clinical trial for castration-resistant prostate cancer [74].

EZH2 must form a functional complex with EED and SUZ12 for epigenetic regulation. Therefore, inhibiting EED or SUZ12 indirectly disrupts EZH2 function. UNC6852, an EED-targeting PROTAC, blocks the histone methyltransferase activity of EZH2, thereby reducing H3K27 trimethylation in DLBCL harboring EZH2 mutations [75]. However, while the oncogenic function of EZH2 represents a promising target for therapeutic intervention, the increased proliferation observed after EZH2 inhibitor treatment and the effects of H3K27 acetylation mediated by EZH2 depletion on the response to EZH2 targeting drugs require further investigation to optimize therapeutic strategies [76].

miRNA modulators which are designed to mimic or inhibit specific miRNAs involved in angiogenesis have been actively researched. MRG-110 targets miR-92a, which negatively regulates angiogenesis. It is currently in preclinical development for wound healing and ischemic conditions, aiming to enhance angiogenesis through epigenetic modulation. Thus, MRG-110 accelerates angiogenesis and wound healing in a diabetic model [77]. Similarly, MRG-201 targets another anti-angiogenic miRNA, miR-29, and exhibits a pro-angiogenic effect for fibrosis and cancer, where modulating angiogenesis is crucial [78]. These drugs represent a promising frontier in the treatment of diseases where aberrant angiogenesis plays a critical role, including cancer, diabetic retinopathy, and cardiovascular diseases. Their ability to modulate the epigenetic landscape offers a novel approach to controlling angiogenesis at the gene expression level.

## Conclusion and future directions

The intricate interplay between angiogenesis and epigenetic regulation is essential for both normal physiological processes and various pathological conditions, particularly in diseases such as cancer, diabetic retinopathy, and atherosclerosis, where angiogenesis plays a pivotal role. This review highlights the significant role of epigenetic modifications, including DNA methylation, histone modifications, and noncoding RNAs, in regulating the expression of key angiogenic factors and pathways. Aberrant epigenetic changes can lead to dysregulated angiogenesis, contributing to disease progression by promoting abnormal blood vessel formation, which supports tumor growth and metastasis, exacerbates inflammation, and impairs tissue repair.

Therapeutically, targeting the epigenetic machinery represents a promising approach for modulating angiogenesis in diseases characterized by abnormal vessel growth. The development of epigenetic drugs, such as DNA methyltransferase inhibitors (e.g., azacitidine, decitabine), histone deacetylase inhibitors (e.g., vorinostat, romidepsin), and bromodomain inhibitors (e.g., JQ1), represents a new frontier in the treatment of angiogenesis-related diseases. These drugs have demonstrated potential in preclinical and clinical studies to inhibit angiogenesis and tumor growth by reactivating silenced tumor suppressor genes and downregulating pro-angiogenic factors.

However, significant challenges remain in the development and application of epigenetic therapies. The specificity of epigenetic drugs is a major concern, as off-target effects can lead to unintended consequences, including the activation of oncogenes or the suppression of essential genes. Moreover, resistance to epigenetic therapies, akin to traditional chemotherapy and targeted therapies, poses a significant hurdle. This necessitates ongoing research to better understand the mechanisms of resistance and to develop combination therapies that can overcome these obstacles.

Looking ahead, several key areas warrant further exploration to fully harness the potential of epigenetic therapies in angiogenesis-related diseases. Emerging technologies, such as CRISPR-based epigenome editing, hold promise for precisely targeting and modifying specific epigenetic marks at disease-relevant loci. This approach could allow for more selective reactivation or silencing of genes involved in angiogenesis, minimizing off-target effects associated with conventional epigenetic drugs. Combining epigenetic drugs with other therapeutic modalities, such as immune checkpoint inhibitors, anti-VEGF therapies, or chemotherapy, holds promise for enhancing therapeutic efficacy and overcome resistance. Investigating optimal combinations and treatment regimens in preclinical models and clinical trials is essential. In addition, identifying reliable epigenetic biomarkers for predicting treatment response and monitoring disease progression remains crucial. This would allow for the stratification of patients who are most likely to benefit from epigenetic therapies, leading to more personalized treatment approaches. While miRNAs and lncRNAs have been implicated in angiogenesis [79], their therapeutic potential remains underexplored. Developing miRNA mimics or inhibitors, as well as lncRNA-targeting therapies, could open novel avenues for modulating angiogenesis in various diseases.

In summary, the field of epigenetic regulation in angiogenesis-related diseases continues to evolve rapidly. By advancing our understanding of epigenetic mechanisms and refining therapeutic strategies, we have significant potential to develop more effective treatments for disease characterized by abnormal angiogenesis.

## Data Availability

No datasets were generated or analysed during the current study.
